# Heritability of Individual Psychotic Experiences Captured by Common Genetic Variants in a Community Sample of Adolescents

**DOI:** 10.1007/s10519-015-9727-5

**Published:** 2015-06-07

**Authors:** Dominika Sieradzka, Robert A. Power, Daniel Freeman, Alastair G. Cardno, Frank Dudbridge, Angelica Ronald

**Affiliations:** Centre for Brain and Cognitive Development, Birkbeck, University of London, 32 Torrington Square, London, WC1E 7HX UK; Medical Research Council Social, Genetic and Developmental Psychiatry Centre, Institute of Psychiatry, Psychology & Neuroscience, King’s College London, London, UK; Department of Psychiatry, University of Oxford, Oxford, UK; Academic Unit of Psychiatry and Behavioural Sciences, University of Leeds, Leeds, UK; Department of Non-communicable Disease Epidemiology, London School of Hygiene and Tropical Medicine, London, UK

**Keywords:** Heritability, Genetics, Schizophrenia, Psychosis, Adolescence, Dimensions

## Abstract

Occurrence of psychotic experiences is common amongst adolescents in the general population. Twin studies suggest that a third to a half of variance in adolescent psychotic experiences is explained by genetic influences. Here we test the extent to which common genetic variants account for some of the twin-based heritability. Psychotic experiences were assessed with the Specific Psychotic Experiences Questionnaire in a community sample of 2152 16-year-olds. Self-reported measures of Paranoia, Hallucinations, Cognitive Disorganization, Grandiosity, Anhedonia, and Parent-rated Negative Symptoms were obtained. Estimates of SNP heritability were derived and compared to the twin heritability estimates from the same sample. Three approaches to genome-wide restricted maximum likelihood (GREML) analyses were compared: (1) standard GREML performed on full genome-wide data; (2) GREML stratified by minor allele frequency (MAF); and (3) GREML performed on pruned data. The standard GREML revealed a significant SNP heritability of 20 % for Anhedonia (SE = 0.12; *p* < 0.046) and an estimate of 19 % for Cognitive Disorganization, which was close to significant (SE = 0.13; *p* < 0.059). Grandiosity and Paranoia showed modest SNP heritability estimates (17 %; SE = 0.13 and 14 %; SE = 0.13, respectively, both n.s.), and zero estimates were found for Hallucinations and Negative Symptoms. The estimates for Anhedonia, Cognitive Disorganization and Grandiosity accounted for approximately half the previously reported twin heritability. SNP heritability estimates from the MAF-stratified approach were mostly consistent with the standard estimates and offered additional information about the distribution of heritability across the MAF range of the SNPs. In contrast, the estimates derived from the pruned data were for the most part not consistent with the other two approaches. It is likely that the difference seen in the pruned estimates was driven by the loss of tagged causal variants, an issue fundamental to this approach. The current results suggest that common genetic variants play a role in the etiology of some adolescent psychotic experiences, however further research on larger samples is desired and the use of MAF-stratified approach recommended.

## Introduction

‘Psychotic experiences’ is an umbrella term for a range of phenomena including paranoia, hallucinations, lack of motivation or inability to experience pleasure. These experiences are common but vary in severity of expression across the population (van Os et al. 2009; Ronald et al. 2014). Individuals can experience individual psychotic experiences with varying frequency and accompanying level of distress (Wigman et al. 2011). Early psychotic experiences have been shown to be a risk marker for later development of a number of psychiatric conditions (Poulton et al. 2000; Kelleher et al. 2012; Fisher et al. 2013).

Similar to adult symptom dimensions in schizophrenia (Fanous and Kendler 2005; Rijsdijk et al. 2011; Fanous et al. 2012) adolescent psychotic experiences show a multidimensional factorial structure (Vollema and Hoijtink 2000; Cyhlarova and Claridge 2005; Fonseca-Pedrero et al. 2009; Ericson et al. 2011; Wigman et al. 2011; Ronald et al. 2014). The number of reported dimensions and their content vary depending on the measure of psychotic experiences used, however it is usually three or more (Cyhlarova and Claridge 2005; Fonseca-Pedrero et al. 2009; Ericson et al. 2011; Wigman et al. 2011; Ronald et al. 2014). Ronald et al. (2014) have identified a six-dimensional solution of paranoia, hallucinations, cognitive disorganization, grandiosity, anhedonia, and negative symptoms, from the same sample as was employed in the current study.

Until recently, little was known about the role of genetic influences on individual psychotic experiences in adolescence (Ronald 2015). Evidence came from twin-based heritability studies, which suggest that the magnitude of the genetic influence on individual psychotic experiences varies depending on dimension (Ericson et al. 2011; Hur et al. 2012; Zavos et al. 2014). The latest twin-based heritability estimates of the above-mentioned six individual psychotic experiences used in the current study have been shown to range from 15 to 59 % (Zavos et al. 2014). Moderate estimates were reported for Negative Symptoms (59 %), Paranoia (50 %), Anhedonia (47 %), Grandiosity (44 %), Cognitive Disorganization (43 %); lower estimates were found for Hallucinations (15 % for males and 32 % for females).

Thanks to recent technological advances, traditional twin-based heritability estimates can be supplemented with estimates of heritability captured by common genetic variants, referred to as SNP-based heritability. This method relies on comparison of matrices of phenotypic and genotypic similarity between unrelated individuals (Yang et al. 2010). SNP heritability provides a lower bound estimate of narrow-sense heritability because it relies on additive genetic effects of common SNPs tagged on microarrays and does not take into account rare or structural genetic variants and gene-environment interactions or correlations. SNP heritability estimates are known to be affected by imperfect tagging of SNPs (Wray et al. 2013), which means that genetic effects of the causal variants that are not in linkage disequilibrium (LD) with the genotyped SNPs are not taken into account in the estimate. As such, the true role of the common genetic variants could be underestimated. The SNP heritability estimates can also be overestimated if causal variants are in strong LD with multiple genotyped SNPs. For these reasons, the effect of LD should be considered when calculating SNP heritability estimates. This can be achieved in a number of ways, including pruning and stratification by minor allele frequency (MAF).

Pruning prevents inflation of the SNP heritability due to LD by removing from the analysis genotyped SNPs in high LD. The main shortfall of this method is that tagging of the causal variants can be lost in the process, thereby underestimating the true SNP heritability. In contrast, stratification by MAF does not suffer from a loss of tagged causal variants. Instead, the MAF-stratified approach uses information about the relationship between LD and MAF to offer an alternative solution to the issue of unequal tagging of SNPs (Wray 2005). LD is typically measured in terms of the squared correlation *r*^2^ between two SNPs, which places a quantifiable constraint on the difference in MAF between two SNPs. In order to achieve an *r*^2^ of at least 0.8 with a SNP with MAF of 0.1 the maximum difference in allelic frequency at the second SNP is ±0.02 (Wray 2005). This results in underrepresentation of causal variants with low MAF by the genotyped SNPs and means that high LD occurs only between SNPs with similar MAF (Wray 2005). The MAF-stratified approach derives estimates of SNP heritability from multiple genetic relationship matrices based on SNPs in different MAF bins thereby providing estimates of heritability that reflect contributions from SNPs based on LD between them (Lee et al. 2013b). The MAF-stratified approach does not assume a constant distribution of effect sizes across the MAFs (Lee et al. 2013b).

The overall importance of common genetic variants in the etiology of psychotic experiences in adolescence is unknown: there are no studies to date of the ‘SNP heritability’ of psychotic experiences in the general population. There has been one genome-wide association (GWA) study on psychotic experiences, which was performed on a single measure of adolescent positive psychotic experiences and no SNPs reached significance post correction for multiple testing (Zammit et al. 2014). Evidence to date shows that common SNPs account for 32 % of the variance in schizophrenia liability (Ripke et al. 2013), for which psychotic experiences are a risk factor.

Here we test the extent to which SNPs account for some of the twin-based heritability estimates of psychotic experiences in adolescence. We compare published twin-based heritability estimates (Zavos et al. 2014) with SNP heritability estimates using individuals drawn from the same sample, assessed at the same age with the same measure. Our aim was to estimate the proportion of variance in individual psychotic experiences in adolescence explained by common genetic variants. Based on SNP heritability estimates from other studies across a range of phenotypes within psychology and psychiatry (Trzaskowski et al. 2013a; Wray et al. 2013; Ripke et al. 2013) we hypothesised that approximately half the twin-based heritability will be accounted for by common genetic variants. In addition to the standard SNP heritability analysis, we applied MAF-stratification and pruning to account for the effects of LD on the SNP heritability estimates.

## Method

### Participants

Participants in the current study were drawn from the Twins Early Development Study (TEDS), a longitudinal general population sample of twins born in England and Wales between 1994 and 1996 (Haworth et al. 2013). Upon initial recruitment, 13,488 families responded with a written consent form. To ensure direct comparison of twin-based and SNP-based heritability estimates, participants were excluded based on the criteria used in the previously published twin study (Zavos et al. 2014) i.e. lack of consent at first contact or for the present study, presence of severe medical disorder(s), lack of zygosity information or experience of severe perinatal complications. Of the recruited children, 4440 provided DNA samples, which were extracted from buccal cheek swabs and sent to Affymetrix Santa Clara, CA, USA to be individually genotyped on the AffymetrixGeneChip 6.0 SNP genotyping platform. This formed part of the TEDS Wellcome Trust Case Control Consortium 2 (WTCCC2) study of reading and mathematical abilities (see http://www.wtccc.org.uk/ccc2/wtccc2_studies.shtml). In total, 3665 samples were successfully hybridized to AffymetrixGeneChip 6.0 SNP genotyping arrays. Of those, 513 were excluded based on low call rate or heterozygosity outliers, atypical population ancestry, sample duplication or relatedness to other sample members, unusual hybridization intensity, and gender mismatches (Trzaskowski et al. 2013b). The final sample of the genotyped individuals was 3152 individuals (1446 males and 1706 females). Phenotypic data on psychotic experiences post exclusions was available for 2152 (931 males and 1221 females) of the genotyped individuals (M = 16.31 years; SD = 0.67 years) and limited to those of white ethnicity. Only participants of white ethnicity were included to ensure the MAFs were consistent across the sample. Table 1 presents a comparison between the current sample and previously published demographic information on TEDS families that provided data at first contact (Haworth et al. 2013).

**Table 1:** Comparison between the current sample and previously published demographic information on TEDS families

	Current sample	All TEDS families^a^
N	2,152	13,694
% male	45	49.9
% MZ	38.3	33.1
% parents with A-levels or higher education	39.9	35.5
% mother employed	47.2	43.1
% father employed	95.1	91.7

N, sample size

^a^All figures were taken from a previously published work by Haworth et al. (2013) and relate to the data collected at first contact

### Measure

#### Specific Psychotic Experiences Questionnaire (SPEQ)

Individual psychotic experiences were assessed using the Specific Psychotic Experiences Questionnaire (SPEQ) (Ronald et al. 2014). The measure comprises five self-report subscales, namely Paranoia (15 items), Hallucinations (9 items), Cognitive Disorganization (11 items), Grandiosity (8 items), Anhedonia (10 items) and one parent-rated subscale of Negative Symptoms (10 items). The SPEQ subscales were derived using principal component analysis and show good to excellent internal consistency (Cronbach’s α ranged from 0.77 to 0.93 in the present sample) and test re-test reliability across a 9-month interval (*r* = 0.65 to 0.74) (Ronald et al. 2014). The SPEQ was validated against one other measure of adolescent positive psychotic experiences, the Psychosis-Like Symptoms questionnaire (PLIKS-Q; Zammit et al. 2011) and showed good agreement with this instrument (Ronald et al. 2014). The SPEQ positive and cognitive psychotic experiences subscales showed significant positive correlations with the PLIKS-Q measure (Hallucinations *r* = 0.60, Paranoia *r* = 0.48, Cognitive Disorganization *r* = 0.41, Grandiosity *r* = 0.27, all *p* < 0.001) (Ronald et al. 2014). Content validity was appraised through clinical expert assessment of the appropriateness of items used for measuring adolescent psychotic experiences (Ronald et al. 2014).

### Statistical analyses

Descriptive statistical analyses were performed in SPSS for Windows (version 18.0).

#### Scale transformation

Due to a moderate skew of some of the SPEQ subscales (Paranoia, Hallucinations, Grandiosity, and Parent-rated Negative Symptoms), the data on these subscales were normalised using van der Waerden’s transformation (Lehman 1975), which converts ranked data to the quantiles of the standard normal distribution.

#### Genome-wide complex trait analysis (GCTA)

Genome-wide restricted maximum likelihood (GREML) analysis performed in the genome-wide complex trait analysis (GCTA) software allows to estimate the heritability of a trait captured by common single nucleotide polymorphisms (SNPs), referred to as SNP heritability. It is achieved by comparing matrices of pairwise genetic and phenotypic similarity across unrelated individuals (Yang et al. 2011). GCTA fits genetic effects as random effects by a mixed linear model in order to derive estimates of variance explained by common SNPs (Yang et al. 2011).

In the current study a genetic relationship matrix (GRM) was first calculated to ensure that the individuals were truly unrelated, and those with a pairwise relatedness of ≥0.025 were removed (this corresponds approximately to a cousin three times removed). Then univariate heritability estimates were obtained using a standard GREML approach (Yang et al. 2011) on the full genome-wide data comprising 1,700,285 SNPs. In addition, to control for the effect of LD two further methods were applied: (1) the analyses were performed on LD-pruned data consisting of 126,984 SNPs (selected using PLINK based on *r*^2^ of 0.25 and sliding window of 200 SNPs); and (2) a MAF-stratified approach was applied to the full genome-wide data (Lee et al. 2013b). Standard GREML and GREML on pruned data approaches attribute more variance to SNPs with lower MAF in order to ensure that on average variance explained by SNPs with lower and higher MAF is equal. This assumes that lower frequency SNPs have a greater effect size than the ones with higher MAF (Lee et al. 2013b). Creation of multiple MAF bins weakens this assumption as it narrows the difference in MAFs and therefore does not assume constant distribution of the effect sizes across the MAF range. We used six MAF bins (<0.05, 0.05–0.1, 0.1–0.2, 0.2–0.3, 0.3–0.4, 0.4–0.5). ‘No constrain’ formulae were applied here across all SPEQ measures and bins in order to obtain accurate sums of SNP heritability estimates from the six bins.

The SPEQ subscales were regressed on age and sex prior to the analyses to facilitate comparison with the published twin heritability estimates. Since the published twin-based heritability estimates showed significant quantitative sex differences on the Hallucinations dimension (Zavos et al. 2014), SNP heritability estimates for this subscale were calculated separately for males and females. To control for population stratification, eight principal components were included as covariates in the model; full details of the principal component analysis are available elsewhere (Trzaskowski et al. 2013b). Finally, quality control required all SNPs used in the GCTA to have a MAF > 0.01; genotyping >0.90; and Hardy–Weinberg Equilibrium *p* > 1 × 10^−6^, which resulted in an inclusion of 1,700,285 out of the total of 1,724,384 SNPs in the final analyses.

## Results

### Descriptive statistics

Descriptive statistics for the individual psychotic experiences as measured by SPEQ are summarised in Table 2.

**Table 2:** Descriptive statistics for the six individual psychotic experiences

	Paranoia	Hallucinations	Cognitive Disorganization	Grandiosity	Anhedonia	Parent-rated Negative Symptoms
N	2133	2138	2133	2136	2134	2140
Mean	11.99	4.54	3.81	5.15	16.03	2.69
Median	10.00	2.00	3.00	4.00	15.00	1.00
Standard deviation	10.18	5.79	2.83	4.22	7.66	3.66
Variance	103.70	33.53	8.00	17.82	58.71	13.36
Observed range	0–71	0–42	0–11	0–24	0–46	0–28

### Genome-wide complex trait analysis (GCTA)

#### Standard genome-wide restricted maximum likelihood (GREML) approach

Table 3 displays SNP heritability estimates of the six SPEQ subscales derived using the standard GREML approach and previously published twin study estimates from the overlapping sample at the same age (Zavos et al. 2014). The GREML results revealed SNP heritability estimates of just under half of the magnitude of the twin-based heritability estimates for Anhedonia and Cognitive Disorganisation. The proportion of phenotypic variation captured by common genetic variants in Anhedonia was 0.20 (SE = 0.12; *p* = 0.046) and in Cognitive Disorganization 0.19 (SE = 0.13; *p* = 0.059). This is in comparison to the twin-based heritability estimates which were 0.47 and 0.43, respectively (Zavos et al. 2014). The point estimate for Grandiosity was 0.17 (SE = 0.13) and for Paranoia 0.14 (SE = 0.13), however SEs were large. This is in comparison to the twin-based estimates of 0.44 and 0.50, respectively (Zavos et al. 2014). The negative estimates for Hallucinations and Parent-rated Negative Symptoms were derived using ‘no-constrain’ formulae and should therefore be interpreted as zero.

**Table 3:** Standard GREML results for the six individual psychotic experiences

	V(G)/V(P)	SE	*p* value	N^a^	Twin h^2b^
Paranoia	0.14	0.13	0.139	2125	0.50
Hallucinations	−0.06 −0.10 (F); −0.30 (M)	0.12 0.23 (F); 0.29 (M)	0.309	2130 1214 (F); 922 (M)	0.32 (F)/0.15 (M)
Cog. Dis.	0.19	0.13	0.059	2125	0.43
Grandiosity	0.17	0.13	0.100	2128	0.44
Anhedonia	0.20	0.12	0.046	2126	0.47
Neg. Symptoms	−0.09	0.12	0.234	2132	0.59

*Cog. Dis.* Cognitive Disorganization, *Neg. Symptoms* Parent-rated Negative Symptoms, *V(G)/V(P)* proportion of the phenotypic variance explained by the common genetic factors, *SE* standard error, *N* sample size, *F* females, *M* males

^a^8 individuals were removed based on GRM cut-off of 0.025, when the analyses were run separately for males and females only two individuals were removed based on the same cut-off, equating to 2136 individuals

^b^twin study heritability estimates as published elsewhere (Zavos et al. 2014)

#### MAF-stratified genome-wide restricted maximum likelihood (GREML) approach

The results of the MAF-stratified analyses are presented in Table 4. Similar to the results from the standard GREML approach, the highest proportion of phenotypic variation captured by common genetic variants was observed in Anhedonia 0.32 (SE = 0.25) and in Cognitive Disorganization 0.23 (SE = 0.25). Consistent with the results from standard GREML, the negative point estimates for the Hallucinations and Parent-rated Negative Symptoms subscales were derived using ‘no-constrain’ formulae and should therefore be interpreted as zero. The point estimates for Grandiosity and Paranoia were 0.10 (SE = 0.25) and 0.06 (SE = 0.25), respectively. All of the MAF-stratified SNP heritability estimates obtained here fall within the SEs of each estimate from the standard GREML analyses. Most of the SNP heritability in the anhedonia subscale was observed in the bin with the lowest MAF < 0.05 (88,437 SNPs) and equated to 0.21 (SE = 0.08).

**Table 4:** GREML results stratified by MAF for the six individual psychotic experiences

	MAF bin	No. of SNPs	V(G)/V(P)	SE	*p* value	N	Twin h^2a^
Paranoia	<0.05	88,437	−0.040	0.08			
	0.05–0.1	168,576	0.012	0.08			
	0.1–0.2	396,922	0.030	0.09			
	0.2–0.3	368,649	−0.056	0.09			
	0.3–0.4	344,955	−0.041	0.08			
	0.4–0.5	332,730	0.157	0.08			
	SUM	1,700,269	0.06	0.25	0.307	2125	0.50
Halluc.	<0.05	88,437	−0.002	0.08			
	0.05–0.1	168,576	0.027	0.08			
	0.1–0.2	396,922	−0.082	0.09			
	0.2–0.3	368,649	0.056	0.09			
	0.3–0.4	344,955	−0.122	0.08			
	0.4–0.5	332,730	0.051	0.08			
	SUM	1,700,269	−0.072	0.25	0.487	2130	0.32 (F)/0.15 (M)
Cog. Disorg.	<0.05	88,437	0.089	0.08			
	0.05–0.1	168,576	0.011	0.08			
	0.1–0.2	396,922	0.013	0.10			
	0.2–0.3	368,649	0.049	0.09			
	0.3–0.4	344,955	−0.015	0.09			
	0.4–0.5	332,730	0.080	0.08			
	SUM	1,700,269	0.23	0.25	0.140	2125	0.43
Grand.	< 0.05	88,437	−0.059	0.08			
	0.05–0.1	168,576	0.015	0.08			
	0.1–0.2	396,922	−0.061	0.09			
	0.2–0.3	368,649	0.082	0.09			
	0.3–0.4	344,955	0.121	0.09			
	0.4–0.5	332,730	0.006	0.08			
	SUM	1,700,269	0.10	0.25	0.219	2128	0.44
Anhedonia	<0.05	88,437	0.214	0.08			
	0.05–0.1	168,576	0.061	0.08			
	0.1–0.2	396,922	−0.131	0.09			
	0.2–0.3	368,649	0.036	0.09			
	0.3–0.4	344,955	0.099	0.09			
	0.4–0.5	332,730	0.040	0.08			
	SUM	1,700,269	0.32	0.25	0.004	2126	0.47
Negative Symptoms	<0.05	88,437	−0.134	0.07			
	0.05–0.1	168,576	−0.051	0.08			
	0.1–0.2	396,922	−0.034	0.09			
	0.2–0.3	368,649	0.047	0.09			
	0.3–0.4	344,955	−0.104	0.08			
	0.4–0.5	332,730	0.046	0.07			
	SUM	1,700,269	−0.23	0.25	0.03	2132	0.59

*Halluc.* Hallucinations, *Cog. Disorg*. Cognitive Disorganization, *Grand*. Grandiosity, *Negative Symptoms* Parent-rated Negative Symptoms, *v(G)/V(P)* proportion of the phenotypic variance explained by the common genetic factors, *SE* standard error, *N* sample size, *F* females, *M* males

^a^Twin study heritability estimates as published elsewhere (Zavos et al. 2014)

#### Genome-wide restricted maximum likelihood (GREML) approach on pruned data

Table 5 presents the results of GREML using pruned data. Two major differences were observed in the results for pruned data compared to the MAF-stratified and standard GREML approaches. Unlike results from the first two approaches, the estimate of SNP heritability for Anhedonia was close to zero (0.007; SE = 0.18). Furthermore, the pruned data gave a high SNP heritability estimate for Grandiosity of 0.41 (SE = 0.19), which accounts for over 90 % of the twin-based heritability estimate of 0.44 (Zavos et al. 2014). In line with the SEs reported for the previous two approaches, GREML on pruned data revealed that the proportion of phenotypic variation captured by common genetic variants for Cognitive Disorganization was 0.32 (SE = 0.18). Also consistent with the results from the previous two approaches, point estimates for the Hallucinations and Parent-rated Negative Symptoms subscales were negative and were derived using ‘no-constrain’ formulae. Again, these estimates should be interpreted as zero.

**Table 5:** GREML results for the pruned data on the six individual psychotic experiences

	Pruned data 126,894 SNPs				Twin h^2b^
	V(G)/V(P)	SE	*p* value	N^a^	
Paranoia	0.03	0.18	0.425	2128	0.50
Hallucinations	−0.08	0.18	0.328	2133	0.32 (F)/0.15 (M)
	−0.07 (F)	0.31 (F)		1215 (F)	
	−0.17 (M)	0.43 (M)		921 (M)	
Cog. Dis.	0.32	0.18	0.041	2128	0.43
Grandiosity	0.41	0.19	0.014	2131	0.44
Anhedonia	0.007	0.18	0.482	2129	0.47
Neg. Symptoms	−0.38	0.18	0.016	2133	0.59

*Cog. Dis.* Cognitive Disorganization, *Neg. Symptoms* Parent-rated Negative Symptoms, *V(G)/V(P)* proportion of the phenotypic variance explained by the common genetic factors, *SE* standard error, *N* sample size, *F* females, *M* males

^a^5 individuals were removed based on GRM cut-off of 0.025, when the analyses were run separately for males and females only two individuals were removed based on the same cut-off

^b^Twin study heritability estimates as published elsewhere (Zavos et al. 2014); when hallucinations are split by sex only 1 individual is removed based on GRM of 0.025

The results of all three approaches are summarised in Table 6.

**Table 6:** Comparison of SNP heritability estimates for the six individual psychotic experiences derived using three different methods

	Standard GREML	MAF-stratified GREML	GREML on pruned data
Paranoia	0.14 (0.13)	0.06 (0.25)	0.03 (0.18)
Hallucinations	−0.06 (0.12)	−0.07 (0.25)	−0.08 (0.18)
Cog. Dis.	0.19 (0.13)	0.23 (0.25)	0.32 (0.18)
Grandiosity	0.17 (0.13)	0.10 (0.25)	0.41 (0.19)
Anhedonia	0.20 (0.12)	0.32 (0.25)	0.007 (0.18)
Negative Symptoms	−0.09 (0.12)	−0.23 (0.25)	−0.38 (0.18)

Standard errors are presented in brackets next to the SNP heritability estimate

No constrain formula was applied to derive accurate estimates from each bin and was extended to the other two methods for better comparison

*Cog. Dis.* Cognitive Disorganization, *Negative Symptoms* Parent-rated Negative Symptoms

## Discussion

This study reveals that common genetic variants play a contributory role in the etiology of some individual psychotic experiences in adolescence. Based on the results of the standard GREML analyses, 20 % of the phenotypic variation in Anhedonia subscale was captured by common SNPs. As expected, this equated to just under half of the twin-based heritability estimate of 47 % (Zavos et al. 2014). The SNP heritability estimate for Anhedonia derived using the MAF-stratified approach was consistent with the estimate from the standard GREML approach. Here SNP heritability was estimated to be 32 % and was mainly driven by the signal from the bin with the lowest MAF (<0.05). This suggests that SNPs with a MAF below 0.05 play an important role in the etiology of anhedonia. At first glance the low SNP heritability estimate for Anhedonia subscale derived from the pruned data approach of 0.7 % stands in contrast to the previous two estimates, however it is plausible that some of the tagged casual variants, particularly those with low MAF, were lost in the pruning process which resulted in a decrease of the SNP heritability estimate noted here.

Common SNPs were also shown to capture between 19 and 32 % of the phenotypic variation in Cognitive Disorganization. The standard GREML generated an estimate of 19 %, followed by 23 % generated from the MAF-stratified approach, with the highest estimate of 32 % derived from the pruned data. It is plausible that in contrast to the Anhedonia results, the SNP heritability estimate derived here from the pruned data is higher due to a more even distribution of genetic variances across MAF spectrum, which can be seen in the MAF-stratified analysis of Cognitive Disorganization compared to Anhedonia. To illustrate this, SNPs with MAF of below 0.05 appear to contribute to SNP heritability of Cognitive Disorganization as much as SNPs with MAF between 0.4 and 0.5 (see Table 4 for details). Similarly to the Anhedonia result, standard GREML estimate of 19 % equates to just under half of the twin-based heritability estimate of 43 % reported for the overlapping sample at the same age (Zavos et al. 2014).

Based on the results of the standard GREML analyses, 17 % of the phenotypic variation in Grandiosity subscale was captured by common SNPs. The estimate dropped to 10 % in the MAF-stratified analysis, which was in contrast to the high estimate of 41 % derived from the analysis on the pruned data. The latter estimate was statistically significant but it was not consistent with the size of the standard errors reported for the standard GREML and the MAF-stratified analyses and as such it is should be treated with caution. Whilst there is some suggestion that common genetic variants might play an important contributory role in the etiology of grandiosity in adolescence, replication in a larger sample is needed.

No significant SNP heritability estimates were found for the remaining three dimensions of the adolescent psychotic experiences (i.e. Paranoia, Hallucinations, and Parent-rated Negative Symptoms). The standard GREML analysis revealed that 14 % of phenotypic variation in Paranoia was captured by common variants but the standard error was large and the estimate dropped to almost zero when the other two approaches were applied. Point estimates for Hallucinations and Parent-rated Negative Symptoms were particularly low, which was consistent across all three approaches. If replicated, these results suggest that detection of common SNPs associated with hallucinations and parent-rated negative symptoms may prove to be more difficult than identifying common SNPs associated with adolescent anhedonia, cognitive disorganization, grandiosity or paranoia because the former appear to have less net variance explained by common SNPs than the latter.

As expected, most of the SNP heritability estimates of psychotic experiences reported here were lower than the previously reported twin-based estimates. These findings are in line with results of other studies from a number of phenotypes such as schizophrenia (Ripke et al. 2013) or cognitive and learning abilities (Trzaskowski et al. 2013a), which typically report no more than half of the twin-based heritability to be accounted by common genetic variants. This is because GREML, unlike the twin method, provides a lower bound estimate of heritability and is affected by an imperfect tagging of SNPs (Wray et al. 2013). In addition, the effect of SNPs is likely to be attenuated due to associations with SNPs rather than with causal variants that are in LD with them. It is plausible that rare and structural genetic variants account for at least some of the remaining twin-based heritability of adolescent psychotic experiences that has not been explained by the common genetic variants.

Our findings suggest that common genetic variants account for approximately half of the twin-based heritability estimates in anhedonia and cognitive disorganization and there is some tentative evidence for their involvement in the etiology of grandiosity and paranoia. As such, if psychotic experiences are considered a risk marker for later development of schizophrenia, these findings provide some evidence for the notion of the psychosis continuum as our findings are consistent with the findings from schizophrenia genetics, which have shown involvement of common SNP in the etiology of this disorder (Lee et al. 2012; Ripke et al. 2013). SNP heritability of clinical psychotic symptoms is currently unknown, however a comparison of such estimates with the current results will help to elucidate further the relationship between individual adolescent psychotic experiences and adult schizophrenia symptoms as and when these become available. The current work is the first step in exploration of the role of common genetic variants in the etiology of individual psychotic experiences in adolescence with a long-term aim of incorporation of molecular genetic findings into early intervention programs aimed at prevention of negative outcomes, such as transitions into clinical diagnosis. However, the focus on the clinical aspects should not overshadow the importance of understanding genetic causes of normal variation in these relatively commonly occurring phenomena in adolescence.

Furthermore, the results for the two measures of negative psychotic experiences (i.e. self-rated Anhedonia and parent-rated Negative Symptoms) deserve discussion. Our results suggested that common genetic variants play a role in the etiology of self-rated Anhedonia but not in Parent-rated Negative Symptoms. These two measures capture different aspects of negative psychotic experiences, and, importantly, rely on different raters (self and parent) which is reflected by the modest correlation of 0.14 (Ronald et al. 2014). The Anhedonia subscale is a reversed measure of hedonia, which assesses anticipatory experience of pleasure (Gard et al. 2006; Ronald et al. 2014) by asking individuals to rate statements such as “Looking forward to a pleasurable experience is in itself pleasurable” (Ronald et al. 2014). In contrast, Parent-rated Negative Symptoms subscale assesses a broader range of negative symptoms: problems with expressing emotions, poverty of speech, lack of motivation, social withdrawal, and problems with attention (Andreasen 1984; Ronald et al. 2014). Negative symptoms were assessed here by asking parents to rate statements in relation to their teenage children, for example “Has a lack of energy and motivation” or “Often does not have much to say for himself/herself” (Ronald et al. 2014). As such, both SPEQ measures capture different types of negative symptoms.

### Comparisons of the three GREML methods

Our results are consistent with previous research, which suggests that estimates derived from standard GREML are largely consistent with those obtained from the MAF-stratified approach (Lee et al. 2012, 2013a, b; Ripke et al. 2013). When the latter approach is used, additional information about the underlying genetic architecture of a trait can be obtained. The MAF-stratified approach allows us to establish if SNPs from a specific MAF bin make a substantial contribution to the overall SNP heritability, like in the case of anhedonia in the current study. The downside of using the MAF-stratified approach is that the increase in the number of estimated parameters (based on a number of MAF bins used) leads to wider SEs and thus requires larger samples for equivalent accuracy in point estimates. Pruning, on the other hand, is a widely used approach suitable for use with smaller samples, however its main drawback is the loss of tagged causal variants in the process of pruning. This in turn may result in a substantial underestimation of the true SNP heritability. As such, whilst each of the approaches has its strengths and limitations, the MAF-stratified approach offers the most detailed information regarding the contribution of SNPs to heritability of a given trait.

### Limitations

The current findings provide new evidence on the genetic etiology of individual adolescent psychotic experiences. However, our study was limited by its sample size, resulting in large standard errors; a larger sample would offer greater accuracy of the point estimates and clearer conclusions about the proportion of twin-based heritability explained. An advantage of this study was that the participants were a subsample of those employed in the study on twin-based heritability. It was not possible to use the exact same sample because genotypic data was available for only a subsample (2152 of unrelated individuals) of the twin sample (4743 twin pairs) (Zavos et al. 2014). Further research in larger samples would allow bivariate GREML analyses to explore the degree to which psychotic experiences show genetic overlap with each other and with related phenotypes in psychopathology. Finally, this study was limited to participants of white ethnicity and as such it is important for this work to be conducted with individuals drawn from other populations.

## Conclusions

This is the first study to show that common genetic variants play a role in the etiology of some individual psychotic experiences in adolescence. Common SNPs accounted for approximately half of the twin-based heritability estimates in anhedonia and cognitive disorganization. There is some evidence that common genetic variants may be important for the genetic architecture of grandiosity and paranoia but further research in larger samples is required. No common genetic effects were found for hallucinations and parent-rated negative symptoms in the current sample. It is hoped that further exploration of the role of common genetic variants in the etiology of adolescent psychotic experiences will help with early intervention and prevention of transitions into clinical diagnosis. Replication of the current findings in a larger sample is desired and the use of the MAF-stratified approach recommended.
